# NK Cell-Microbiota Interaction Biomarker Strategy: Advancing Prostate Cancer Management

**DOI:** 10.3390/biom15020273

**Published:** 2025-02-13

**Authors:** Sara Fanijavadi, Torben Frøstrup Hansen, Ahmed Hussein Zedan

**Affiliations:** 1 Cancer Polyclinic, Levanger Hospital, 7601 Levanger, Norway; 2 Department of Oncology, Vejle Hospital, University Hospital of Southern Denmark, 7100 Vejle, Denmark; 3 Department of Oncology, Institute of Regional Health Research, University of Southern Denmark, 7100 Vejle, Denmark

**Keywords:** prostate cancer, biomarker, NK cells, microbiota

## Abstract

The role of natural killer (NK) cells in the management of prostate cancer (PCa) remains incompletely understood. Some have proposed that measuring NK cells in blood samples could serve as a reliable, minimally invasive tool for screening, assessing treatment effects, and predicting survival outcomes in PCa patients. However, the significance of different NK cell phenotypes remains unclear. Given the interplay between NK cells and the microbiome, we hypothesize that a combined signature of NK cell phenotypes derived from blood, along with microbiome profiles from oral, urine, and stool samples, could serve as a surrogate marker for NK cell activity in tumor and its microenvironment. Such an approach provides a practical alternative to invasive tumor biopsies by enabling the indirect assessment of NK cell function in tumors. Additionally, profiling NK cell phenotypes and their interactions with the microbiota has the potential to enhance prognostic accuracy and guide the development of personalized therapeutic strategies. Prospective studies are needed to validate the utility of NK cell and microbiome assays in personalized PCa management, with a focus on minimally invasive procedures and predictive signatures for treatment outcomes.

## 1. Introduction

Prostate Cancer (PCa) is the second most common noncutaneous neoplasm and the fifth most common cause of cancer-related deaths in men worldwide [1]. PCa is a heterogeneous disease, with most cases progressing slowly [2], but the prognosis can vary significantly. Early detection and monitoring of PCa are critical for effective management. Measurement of serum Prostate-Specific Antigen (PSA) is the cornerstone of current diagnostic practices [3]. Since its introduction in the late 1960s, PSA testing has dramatically shifted the staging of PCa, with most cases diagnosed as prostate-confined tumors [4]. Despite its widespread use, the optimal cut-off for elevated PSA remains debated [5]. Using a threshold of 4 ng/mL, the positive predictive value of PSA in diagnosing PCa varies widely from 25% to 40% [6], with a sensitivity and specificity of around 20% and 65% [7].

While PSA testing has increased early detection, it has also led to overdiagnosis, contributing to a rise in PCa incidence, particularly in the mid-1980s. Recent recommendations for more conservative PSA testing have reduced the diagnosis of new cases [8]. The conventional diagnostic tool for PCa is Transrectal Ultrasound (TRUS)-guided biopsy [9], but it has limitations, including invasiveness, side effects, and a risk of false-negative results [10]. To improve diagnostic accuracy, Magnetic Resonance Imaging (MRI)-targeted biopsy is being explored as a more effective alternative [11].

The risk of recurrence after curative management of PCa primarily depends on the disease stage at initial diagnosis, which is typically determined by clinical Tumor, Node, and Metastasis (TNM) staging, PSA levels, and biopsy Gleason Score (GS) [2]. Radical prostatectomy and radiotherapy are the primary treatments for localized and locally advanced PCa [12], while Androgen Deprivation Therapy (ADT) remains the standard medical treatment for both high-risk localized PCa and metastatic disease [13]. Unfortunately, nearly all patients with incurable PCa eventually progress to the Castration-Resistant. Prostate Cancer (CRPC) stage, with 35% developing metastases and a survival rate of just 2–4 years post-progression [14].

Accumulating evidence suggests that impaired immune response plays a critical role in the pathogenesis of PCa [15,16]. Both preclinical and clinical studies have highlighted the involvement of inflammation in PCa development and progression [17,18]. Immune biomarkers are being increasingly recognized as valuable tools for the early diagnosis and management of various cancers, including PCa [19,20]. Understanding the role of the immune system, particularly the innate immune system, is crucial for improving therapeutic outcomes.

The immune system’s defense mechanisms are primarily mediated by the innate and acquired immune systems, which distinguish between “self” and “non-self” antigens [21]. Natural Killer (NK) cells are a key component of the innate immune response and differ from B and T lymphocytes. Representing 10–15% of all circulating lymphocytes, NK cells are identified by the absence of CD3 and the presence of CD56 [22,23]. NK cells are found throughout the tumor stroma and around the glandular epithelium, indicating their potential involvement in both tumor progression and the immune response [24]. However, inflammation in the tumor microenvironment (TME) can impair NK cell function, reducing their anti-tumor activity.

**Figure 1:**
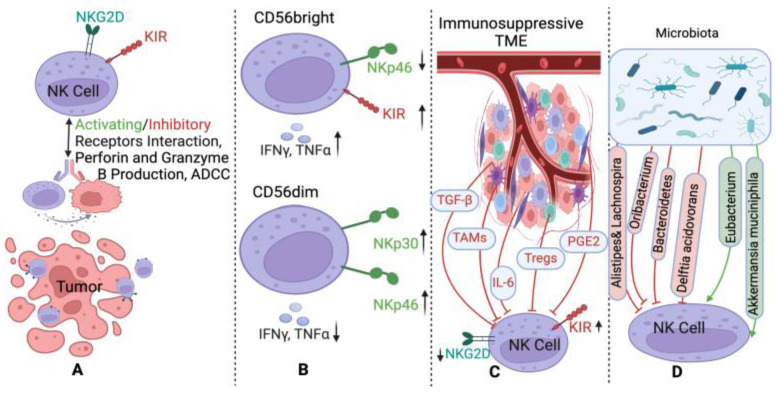
NK Cell Function, Regulation, and Clinical Implications in Prostate Cancer (PCa). The figure uses different arrow types to represent various interactions: a green arrow indicates activation, a red arrow represents inhibition, a black double-headed arrow signifies interaction, an upward black arrow denotes an increase, and a downward black arrow signifies a decrease. (**A**) NK Cell Function: NK cell activity is regulated by a balance between activating receptors such as NK Group 2 Member D (NKG2D) and inhibitory receptors like Killer-cell Immunoglobulin-like Receptor (KIR). Their function includes direct cytotoxicity through granzyme B and perforin production, as well as antibody-dependent cellular cytotoxicity (ADCC). (**B**) NK Cell Subsets: Shows the two main subsets: CD56^dim^CD16^+^ (cytotoxic) and CD56^brigh^CD16^−^ (cytokine-producing), which can become cytotoxic as well. (**C**) Tumor Microenvironment (TME) and NK Cell Suppression: Depicts how factors in the TME like tumor growth factor β (TGF-β), interleukin 6 (IL-6), Regulatory T cells (Tregs), prostaglandin E2 (PGE2), and tumor-associated macrophages (TAMs) suppress NK cell function. (**D**) Microbiome Influence on NK Cells: Shows how different bacteria in the microbiome can either support or suppress NK cell activity in PCa (Biorender, Fanijavadi, S. (2025) https://BioRender.com/a43k204 (accessed on 7 February 2025)).

NK cells are classified into two subsets: CD56^dim^ CD16^+^ (cytotoxic) and CD56^brigh^CD16^−^ (cytokine producers). The latter is less mature but can differentiate into CD56^dim^ cells with strong cytotoxic activity [21,25]. NK cells recognize tumor cells by detecting low or absent class I Human Leukocyte Antigen (HLA) molecules and lysing them directly. They also bind NK recognition structures on tumor cells, releasing NK Cytotoxic Factor (NKCF) to induce tumor cell lysis [26]. NK cell activity also depends on the recycling capacity of NK cells after lysis of the tumor cell and can be augmented by interferons (IFNs) [22]. Their activity is influenced by a balance of activating (e.g., NKG2D) and inhibitory (e.g., Killer Immunoglobulin-like Receptors, KIR) receptors, which interact with stress ligands or Major Histocompatibility Complex (MHC) class I molecules [18,25,27,28,29], (Figure 1A,B).

In addition to the direct cytotoxicity mediated by perforin and granzymes, NK cells can also kill target cells through Antibody-Dependent Cell-Mediated Cytotoxicity (ADCC). This occurs when NK cells, through their CD16 receptors, bind to antibody-coated tumor cells, triggering granule release and tumor lysis [15,30] (Figure 1, section A).

NK cell function is enhanced by cytokines such as interleukin-2 (IL-2) and IFNs but can be suppressed by Transforming Growth Factor-beta (TGF-β), suppressor T cells, monocytes, and prostaglandins, which downregulate activating receptors and impair cytotoxicity [31,32], (Figure 1, section C).

In PCa, NK cell activity has potential diagnostic and prognostic value, complementing PSA testing [13,15,33]. The immune response, including NK cell function, can also be influenced by therapies such as ADT, which increase the number of circulating lymphocytes, including NK cells [34]. However, for better diagnostic accuracy, NK cell activity in blood samples must be matched with the phenotype of infiltrating NK cells in tumors.

Despite their potential, relying solely on NK cell biomarkers has limitations, particularly the challenge of monitoring phenotypic changes during treatment via biopsies. To overcome this, combining NK cell biomarkers with additional markers—such as microbiome profiles from oral, stool and urine samples—could facilitate the design of large-scale prospective studies to identify a combinatorial signature of NK cells and the microbiome. Recent studies have underscored the important role of the microbiota in PCa pathogenesis [35], highlighting how specific microorganisms in Pca tissue, such as Vibrio parahaemolyticus and Shewanella, can alter immune responses, potentially aiding tumor evasion [36]. Additionally, the presence of microbes like Delftia acidovorans and Gardnerella vaginalis is linked to the down-regulation of immune-related genes, suggesting an immune-suppressive TME in PCa [37].

Given that NK cells interact with the microbiome (Figure 1, section D), we hypothesize that a combined signature of NK cell phenotypes and microbiome profiles from stool, urine and oral samples could serve as a surrogate marker for NK cell activity. This approach offers a feasible alternative to directly monitoring NK cell phenotyping through biopsies, enabling indirect assessment of NK cell function in tumors through NK cell activity in blood tests and microbiome signatures. The role of NK cells in the management of PCa remains incompletely understood. Measuring NK cells in blood samples has been proposed as a reliable, minimally invasive tool for screening, assessing treatment effects, and predicting survival outcomes in PCa patients. This review explores current evidence on the potential role of NK cells in PCa management, with a particular focus on the interaction between NK cells and the microbiome.

## 2. NK Cells and PCa

### 2.1. NK Cells: Pathogenesis Significance in PCa

NK cells play a critical role in the innate immune response against PCa by identifying and eliminating tumor cells. Their function, however, is influenced by the TME. NK cells interact with other immune cells, including cytotoxic T lymphocytes (CTLs) and natural killer T (NKT) cells, through cytokine signaling that enhances immune responses against the tumor. This cooperation between innate and adaptive immune cells is important for immune surveillance but can be disrupted by factors in the TME, leading to immune evasion [38]. Several studies have highlighted that the suppression of NK cell function, both at the neoplastic and pre-neoplastic stages of tumorigenesis, as well as during the induction and progression of cancer, plays a critical role in disease development [39].

However, in this review, we specifically focus on the interactions between NK cells and the microbiome rather than their interactions with adaptive immune cells such as CTLs and NKT cells. We hypothesize that the NK cell phenotype is the key determinant of immune outcomes. Here, we highlight the three main mechanisms of NK cell dysfunction, as previously described [40]: (1) impaired NK cell proliferation, (2) reduced cytotoxicity, and (3) decreased tumor infiltration.

#### 2.1.1. Impaired Proliferation of NK Cells

Impaired proliferation is one of the most well-studied mechanisms, as highlighted by numerous studies. However, this mechanism can overlap with the other two since a reduced frequency of NK cells in peripheral blood often correlates with decreased NK cell activity [3,15,41]. For example, a lower frequency of NK cells can result in decreased production of IFN-*γ*, a key marker of NK cell cytotoxic activity. However, it’s important to note that NK cell activity in peripheral blood may not necessarily correspond to the same function or phenotype within the tumor and TME [41]. Therefore, it is essential to measure both peripheral blood NK cells and tumor-infiltrating NK cells (preferably simultaneously) to accurately assess their true correlation. Only by doing so can we determine whether blood tests can serve as reliable surrogates for the actual function of NK cells in the tumor and TME.

Elevated NK cell expression within tumors has been linked to a lower risk of recurrence, suggesting that NK cells play a protective role in controlling tumor growth [42,43,44]. A reduction in the frequency of the cytotoxic CD56^dim^ NK cell population—both in peripheral blood and within tumors—appears to be a critical factor in cancer progression. While Sotosec et al. found no significant differences in NK cell percentages or subsets in peripheral blood between patients with localized, locally advanced, or metastatic PCa, Koo et al. demonstrated a significantly higher CD56^dim^-to-CD56^bright^ ratio in the peripheral blood of PCa patients compared to healthy controls. This ratio increased progressively with the cancer stage [3,15].

The impact of cancer therapies on NK cell proliferation has been explored in several studies. For example, androgen deprivation therapy (ADT), or low testosterone levels associated with ADT, may contribute to decreased NK cell counts in peripheral blood, potentially indicating a shift of NK cells from circulation to tumor sites [45,46,47]. This could impair NK cell function in peripheral blood but increase NK cell infiltration into the tumor and TME [48,49,50]. In fact, PCa patients undergoing ADT have shown higher IFN-*γ* expression. Perforin production was lower in the PCa group compared to the control, which can be attributed to the low activity of NK cells and low perforin production in cancerous tissues compared to healthy control.

Radiotherapy reduces leukocyte and lymphocyte counts in peripheral blood [51], but it may also alter the TME to become more favorable for NK cells, potentially enhancing their infiltration into solid tumors, including PCa [52]. Docetaxel, a chemotherapy agent, has been shown to enhance NK cell activity and may promote NK cell proliferation through immune signaling pathways like cGAS/STING-IFN. However, the effect of docetaxel on NK cell infiltration into tumors is less clear, and changes in the TME may influence tumor infiltration indirectly [53].

**Figure 2:**
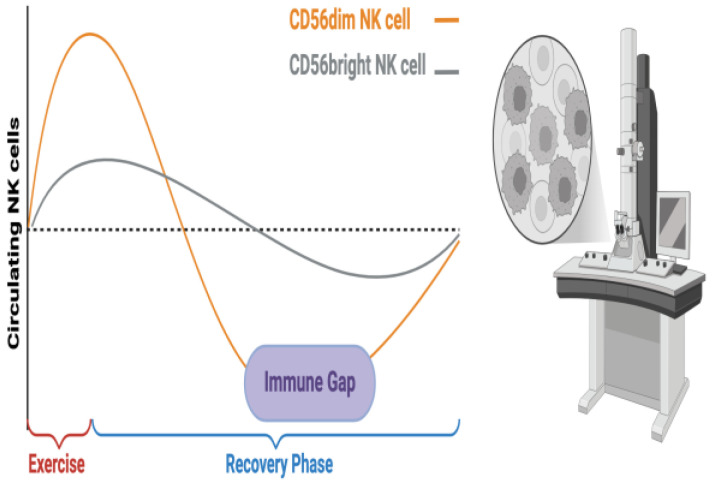
Exercise-Induced NK Cell Mobilization and the Immune Gap. This diagram shows how NK cells increase during exercise but drop sharply post-exercise under the normal baseline level (dotted line), creating an immune gap—a temporary period of reduced immune surveillance. CD56^dim^ NK cells, which are more responsive to exercise, show a greater increase and decline compared to CD56^bright^ NK cells. This drop may reduce tumor infiltration, weaken cytokine signaling, and potentially allow tumor cells to evade detection response (Biorender, Fanijavadi, S. (2025) https://BioRender.com/j96q812 (accessed on 7 February 2025)).

The mobilization and egress of NK cells during exercise have also been extensively discussed in the literature [49,51]. Although acute exercise induces NK cell mobilization into circulation, lymphocyte numbers tend to decline sharply during the first few hours of recovery [54,55,56,57]. Interestingly, CD56^bright^ cells, a less mature and less frequent NK subset primarily responsible for cytokine production, show a smaller response to exercise compared to their CD56^dim^ counterparts [58]. Studies on PCa survivors have confirmed that acute exercise induces a temporary increase in NK cell numbers [59,60,61,62]. However, the subsequent sharp decline in NK cell counts during the recovery phase raises concerns about the potential long-term impact of this reduction, such as increased susceptibility to infections or diminished NK cell function. In addition, these fluctuations in NK cell numbers may influence immune cell infiltration and tumor viability [62,63,64] (Figure 2).

#### 2.1.2. Decreased Cytotoxicity

CD56^dim^ NK cells, particularly those expressing CD57 (CD57^+^) (a marker of NK cell maturation), are the primary subset responsible for cytotoxic function. These cells exhibit greater cytotoxic activity than CD57^−^ NK cells and have a higher capacity for mobilization and egress compared to other NK cell subsets [64,65,66]. The PCa TME is characterized by an enrichment of exhausted CD56^dim^ NK cells. These NK cells, despite their cytotoxic potential, often exhibit reduced activity due to a combination of immune evasion mechanisms, including the dysregulation of activating and inhibitory receptors [67]. Additionally, NK cells produce IFN-*γ* upon activation, which may further enhance their cytotoxic function [56,68]. However, in PCa, immune escape mechanisms are often linked to the low killing capability of NK cells. For example, the Hypoxia-Inducible Factor 1 (HIF-1*α*) pathway can inhibit the NCR1/NKp46 signaling pathway via miR-224, reducing NK cell activity [69]. Koo et al. (2013) found that NK cell activity was much higher in healthy individuals (975.2 ± SD pg/mL) than in PCa patients (430.9 ± SD pg/mL), indicating a significant decline in NK cell function with cancer. This reduction may weaken immune surveillance and promote tumor progression in PCa patients [15].

A study of peripheral blood NK cells from PCa patients revealed an exhausted NK phenotype with significantly reduced cytotoxicity. These exhausted NK cells release cytokines and chemokines that recruit monocytes, which then polarize into M2-like tumor-associated macrophages (TAMs). These TAMs promote angiogenesis and immune suppression within the TME, facilitating further immune evasion by the tumor. Profiling peripheral NK cells in PCa patients could provide valuable insights into immune dysfunction and help inform the development of targeted therapies [69,70].

Another key mechanism contributing to NK cell dysfunction in PCa is the downregulation of NKG2D, an activating receptor crucial for NK cell-mediated tumor recognition. Gallazi et al. proposed that downregulation of NKG2D on circulating NK cells in patients with localized or locally advanced PCa is associated with increased markers of NK cell exhaustion, such as PD-1 and Tim-3 [70]. Moreover, Lundholm et al. demonstrated significant downregulation of NKG2D on circulating NK cells in castration-resistant prostate cancer (CRPC) patients compared to healthy controls [71]. In de novo metastatic PCa, tumor recognition by NK cells involves the activation of NKp46, NKG2D, and NKp30 receptors [45]. However, in patients with advanced PCa, the maximal cytotoxic potential and recycling capacity of NK cells are reduced, leading to suppressed NK cell activity [72]. Additionally, cytokine dysregulation is thought to contribute to reduced NK cell cytotoxicity in advanced PCa. Lahat’s group found that patients with advanced PCa exhibited the lowest NK cell activity and IL-2 secretion compared to those with localized PCa and healthy controls [73]. The high levels of soluble MICA (sMICA) observed in advanced PCa patients were linked to reduced NKA [74].

Inhibitory receptors, such as KIRs, may also contribute to immune escape by limiting NK cell cytotoxicity. While some studies have suggested a link between KIR gene expression and various cancers, including hepatocellular carcinoma [75] and bladder cancer [76], Portela et al. found no significant difference in KIR and HLA gene frequencies between healthy controls and untreated localized PCa patients [77]. This suggests that KIRs may not play a major role in immune evasion in localized PCa, though they may be relevant in other cancer types or advanced stages.

Interestingly, a preclinical study has highlighted an unexpected role for NK cells in the PCa TME. It showed that NK cells preferentially migrate to CRPC cells rather than normal prostate cells and may suppress the expression of androgen receptor splicing variant 7 (ARv7), a marker of enzalutamide resistance. This suppression leads to reduced CRPC cell growth and invasion [78]. NK cell adoptive transfer therapy could, therefore, represent an effective treatment for enzalutamide-resistant CRPC.

TGF*β*1 and prostaglandin E2 (PGE2) are known to drive NK cell exhaustion, which further dampens immune responses. Additionally, dendritic cells (DCs) can interact with NK cells and play a key role in modulating their function in cancer. DCs can enhance NK cell cytotoxicity, improving their ability to eliminate tumor cells [79]. As a result, DCs are being explored as a therapeutic target, with ongoing efforts to develop autologous DC-based immunotherapies to boost NK cell activity and improve tumor eradication [80].

Several studies have reviewed the mechanisms underlying tumor cell immune escape and NK cell dysfunction within the PCa TME (Table 1). Despite the use of varying methodologies, these studies consistently found reduced NK cell activity in PCa patients, with all agreeing that low NK cell activity is associated with higher tumor incidence and metastasis. These findings suggest that enhancing NK cell activity could serve as a potential adjuvant therapy to prevent relapse, either as a standalone treatment or in combination with other cancer therapies to inhibit disease progression.

A better understanding of the detailed mechanisms behind NK cell dysfunction in the PCa TME may be crucial in developing PCa-specific NK cell-based therapies or prophylactic strategies to improve clinical outcomes.

#### 2.1.3. Diminished Tumor Infiltration

There is some debate regarding the clinical significance of immune cell infiltration in PCa, particularly the role of different NK cell subpopulations in tumor tissue. For example, mononuclear cells from peri-prostatic lymph nodes in PCa patients—whether localized, locally advanced, or metastatic—showed significantly lower reactivation by beta-interferon stimulation compared to those from healthy subjects or autologous peripheral mononuclear cells [81]. These findings suggest that the PCa TME may impair NK cell activity by altering the composition of NK cell subpopulations. Furthermore, while the physical disruption of the tumor capsule possibly leads to tumor invasion in patients with localized and advanced PCa, this theory is still debated [82]. Even within an activating NK cell subset, there is a lack of consensus on the impact of NK cell tumor infiltration.

In 2016, Pasero et al. reported that NK cell infiltration into localized and metastatic PCa tissues was primarily driven by CD56/Neural Cell Adhesion Molecule 1 (NCAM1)-positive NK cells, a phenotype associated with low or absent cytotoxic potential [18]. This raises further questions about the functional role of NK cell infiltration in the PCa TME.

In the context of immune suppression, the role of M2 TAMs is notable. These macrophages promote tumor growth and metastasis by suppressing NK cell activity in the TME and supporting new blood vessel formation. They are driven by cytokines like IL-6, IL-10, and TGF-β, especially in hypoxic conditions or when IL-4 levels are high. M2 TAMs release factors such as TGF-β and IL-10 to aid their own maturation. Additionally, non-coding RNAs and transcription factors, such as c-Myc, contribute to M2 activation and stimulate cancer-associated fibroblasts (CAFs), affecting NK cell infiltration [83,84].

Moreover, combining ADT with exercise has shown promise in improving NK cell infiltration and reducing tumor volume in PCa [85]. This finding highlights the potential of lifestyle interventions in modulating immune responses within the TME.

### 2.2. NK Cells: Screening/Diagnosis Significance in PCa

Accurate risk prediction is a major clinical challenge in PCa management, as it is critical for determining the most appropriate treatment approach. While men with low-risk PCa may not require active treatment, the mere diagnosis of the disease can have significant social, financial, and psychological implications. Currently, the PSA test is the primary screening tool, but it lacks accuracy and reliability, often leading to unnecessary biopsies. As a result, there is a growing need for alternative, non-invasive approaches—such as circulating biomarkers—that offer greater specificity and sensitivity (Table 2).

Recent studies suggest that the detection of NK cells in peripheral blood could play a role in the early diagnosis of PCa. For example, Barkin et al. observed in a small cohort of 43 patients that those with lower levels of NK cells were more likely to have a positive prostate biopsy result [86]. Additionally, recent findings indicate that peripheral NK cells in PCa patients are associated with enhanced expression of markers like CD9, CD49a, CXCR4, CXCL8, and MMP-9. These factors are involved in monocyte recruitment and polarization, highlighting their potential role in PCa diagnosis [70].

**Table 2:** NK cell and diagnosis/Screening.

Year/Reference	No/Population	Sample/Method	Main Finding
2020/[87]	41/Asymptomatic PCa (PSA < 20 ng/mL) 31/Control (Benign biopsies)	PB/Flow cytometric profiling combined to Machine learning	NK cell profiling identified eight distinct features that differentiate PCa from benign disease, making it a potential screening tool for prostate cancer detection.
2020/[88]	18/PCa, GS > 7 8/PCa, GS = 7 24/PCa, GS = 6 52/PCa, Benign	PB/NK Vue	NKA demonstrated a sensitivity of 68% and a specificity of 73% for detecting PCa, with a cut-off value of 500 pg/mL.
2019/[89]	25/Localized PCa 37/Local advanced, advanced 32/Not detected in biopsies.	PB/NK Vue	Low NKA can serve as a predictive tool for positive PCa biopsy, with a cut-off value of 200 pg/mL
2018/[90]	221/Pca 18/GS 9–10 25/GS 8 49/GS 7 43/GS 6 86/Biopsy negative	PB/NK Vue	The study could not confirm the usefulness of NKA for detecting PCa or predicting the Gleason grade.
2017/[91]	21/PCa 22/Negative Biopsy	PB, Tissue/NK Vue	The study’s researchers found that the absolute risk of PCa is 86% when NKA is low.
2014/[18]	8/mPCA 36/mBC 30/mCRC	PB/ Flow Cytometry	High levels of circulating tumor cells (CTCs) are associated with low NKA levels.
2013/[14]	51/PCa 8/GS = 9 8/GS = 8 25/GS = 7 10/GS = 6 54/Negative Biopsy	PB/NK Vue	NKA demonstrates a sensitivity of 72% and specificity of 74% for detecting PCa, with a cut-off value of 530.9 pg/mL.
2004/[73]	23/PCa 10/Healthy control	Serum/ELISA	sMIC is proposed as a novel biomarker for the detection of high-grade PCa.
1993/[86]	23/Localized and advanced PCa 10/Healthy control 6/Chronic diseases	PB/51-Cr	NK lytic activity in patients with untreated PCa effectively differentiates between tumor dissemination and localized PCa.

Prostate Cancer (PCa), Metastatic Breast Cancer (mBC), Metastatic Colorectal Cancer (mCRC), Peripheral Blood (PB), Gleason sum (GS); Natural Killer Activity (NKA), circulating tumor cells (CTCs).

Furthermore, NK cell assays could serve as valuable tools for detecting residual microscopic disease after prostatectomy, potentially informing personalized adjuvant treatment strategies for high-risk patients. Specific markers such as low IFN-*γ* levels, decreased CD56^bright^ NK cells, high CD56^dim^-to-CD56^bright^ NK cell ratios, and elevated sMIC may indicate a higher risk of residual disease. Monitoring NK cell activity during radiotherapy could help ensure that NK cell activity does not decrease, which could otherwise contribute to disease progression.

#### 2.2.1. Radionuclide Labeling Method

Some studies using the 51Cr release assay have suggested that NK cells could serve as biomarkers for both the diagnosis and staging of PCa [82,87]. One 1993 study reported a sharp decline in NK cell activity in patients with tumor lesions in lymph nodes, bones, or soft tissues, although tumor differentiation had no impact on these results. The study proposed that measuring NK cell lytic capacity could supplement routine clinical staging [87].

#### 2.2.2. Flow Cytometry and Machine Learning

A computerized model based on profiling NK cell subsets in the blood of 72 asymptomatic men with PSA levels below 20 ng/mL showed that certain NK cell markers, such as CD56^dim^ CD56^bright^, CD56+ NKp30+, and CD56+ NKp46+, may help detect the presence of PCa. Furthermore, NK cell profiling demonstrated the ability to differentiate between low, intermediate, and high-risk PCa by analyzing 32 phenotypic features [88].

#### 2.2.3. NK Vue Cytokine Release Method

A 2013 study found NK cell activity to be a potential diagnostic marker for PCa with a sensitivity of 72% and specificity of 74%. The CD56^dim^-to-CD56^bright^ cell ratio showed a sensitivity of 66% and specificity of 71% [15,89]. Additional findings supported NK cell activity as a predictor of positive biopsy results, with patients exhibiting low NK cell activity having five times the risk of biopsy-verified PCa [91]. A pilot study indicated that patients with NKA levels below 200 pg/mL had an 86% risk of having PCa [86]. However, a cross-sectional study questioned the utility of NK cell activity for detecting PCa or predicting Gleason scores, noting that serial NK cell activity measurements were needed for more conclusive assessments [90,92].

#### 2.2.4. Secretome Analysis

Unlike the NK Vue test, secretome analysis offers a broader perspective on NK cell behavior by identifying a range of secreted factors that influence tumor progression and immune responses. For example, Gallazzi et al. demonstrated that NK cells in the PCa TME release pro-inflammatory cytokines and chemokines that recruit monocytes and promote M2-like polarization, which may facilitate immune suppression and tumor growth [70].

#### 2.2.5. MICA ELISA

One study found elevated levels of soluble MICA (sMICA, 21 ng/mL) in the serum of nearly all PCa patients with a Gleason score ≥ 6, suggesting that sMICA could serve as a novel biomarker for PCa [74]. NK cell assays, including those measuring sMICA, may complement routine clinical management of PCa, although methods like 51Cr-release assays are difficult to manage due to their reliance on radioisotopes. Alternative methods like the ATP assay or flow cytometry-based assays offer more practical ways to assess NK cell activity, with the ATP assay measuring both NK cell activity and other cytotoxic activities [20,93].

#### 2.2.6. Molecular Profiling

A comprehensive molecular profiling study, including whole-exome sequencing (WES) and whole-transcriptome sequencing (WTS), involved 3365 PCa samples. In prostate biopsies, higher NK cells were less common with certain mutations and AR-V7 but more common with immune suppressors, suggesting an anti-inflammatory response [94]. Recent advances in single-cell RNA sequencing have revealed a variety of innate immune cells in normal prostate tissue, with transcriptional changes observed in PCa. Notably, CD16^−^ NK cell signatures and CCL5 transcripts are found together in the TME, suggesting that these resident-like NK cells may recruit tumor antigens recruiting cells and potentially improve treatment responses [95].

### 2.3. NK Cells: Prognostic and Predictive Significance in PCa

The prognostic and predictive roles of NK cells in PCa have been widely investigated, with the potential to improve personalized treatment strategies, including the use of adjuvant therapies. This section reviews the findings from multiple studies assessing the significance of NK cells as both prognostic and predictive biomarkers in PCa (Table 3).

#### 2.3.1. NK Cells and Prognostic Significance

Gannon et al. found that a high number of CD56^+^ NK cells in localized PCa were linked to a lower risk of disease progression [24]. Similarly, Pasero et al. showed that increased CD56^+^ NK cells in prostate tumors correlated with reduced tumor invasion in seminal vesicles, translating to improved survival outcomes. Specifically, patients with high NKp30 expression had a 3-year survival rate of 85%, compared to just 38% in those with low NKp30 expression [45].

Other studies further support this finding. For example, research from 1995 highlighted an association between high expression of HNK-1 antigen in well-differentiated PCa and better prognosis [33]. Moreover, radiotherapy-induced gastrointestinal (GI) side effects appeared to improve prognosis by stimulating the release of Heat Shock Proteins (HSPs), which augment immune function and enhance NK cell cytotoxicity [97].

In contrast, lower NK cell activity has been linked to more advanced disease stages. Marumo et al. reported significantly lower NK cell activity in advanced PCa patients compared to localized cases and healthy controls [72]. Similarly, Lu et al. demonstrated that postoperative NK cell activity was higher in lower-stage PCa patients and those with negative surgical margins, suggesting NK cell activity as a marker of better post-surgical outcomes [102]. However, Blomgren et al. found that post-radiotherapy NK cell activity was lower than pre-radiotherapy levels, potentially contributing to disease progression [100].

Moreover, several studies have noted an increased ratio of CD56^dim^-to-CD56^bright^ NK cells in the blood of patients with more advanced PCa stages, further supporting the link between NK cell function and disease severity [15]. In metastatic cancer, a significant reduction in NK cell cytotoxicity has been observed, correlating with poor prognosis, especially in patients with high circulating tumor cells (CTCs) [19].

Therapeutic interventions can also affect NK cell activity. A Phase I trial combining ipilimumab and PROSTVAC immunotherapy showed an increase in NK cell subsets expressing Tim-3+, correlating with improved survival [101]. However, a study involving ADT found no significant correlation between IL-2 levels (an NK cell activator) and progression-free survival (PFS) in metastatic PCa, suggesting that NK cell activity is not always predictive of overall survivor (OS) [98], despite ADT induced CD56^+^ increased NK cells in PCa, was associated with good prognosis [24].

Moreover, NK cell markers such as NKp46 have been identified as predictive of OS and time to castration resistance (TCR) [45]. A study monitoring serial serum NK cell activity during treatment of mCRPC found a significantly lower response rate and shorter PFS in patients with IFN-*γ* levels below 200 pg/mL [98].

Recent studies also support the role of NK cells in predicting OS across multiple cancer types, including PCa. Higher NK cell infiltration has been linked to improved OS, particularly in tumors with negative immune regulators, such as AR-V7 (a biomarker of resistance to androgen axis-targeted therapies) [94]. Elevated NK cell expression in prostate tumors is also associated with a lower risk of biochemical recurrence after radical prostatectomy [24,45].

However, NK cells infiltrating prostate tissues often display immature characteristics, with decreased expression of activating receptors such as NKG2D and CD16. This phenotypic immaturity may be mediated by factors like TGF-*β*1 and PGE2, which impair NK cell function, reducing their cytotoxic potential [18].

Furthermore, the expression of various NK cell subpopulations, such as CD56^dim^ has been shown to correlate with exhaustion and poor prognosis [17]. In peripheral blood, high monocyte counts have been linked to aggressive disease features and poor survival in patients with CRPC, although this association is less clear in earlier disease stages [103,104,105,106].

Interactional factors affecting NK cell activity within the TME have also been shown to be associated with prognosis in many studies [99].

NK cell markers have also been linked to prognosis in patients undergoing ADT. NK cells from metastatic PCa patients who show a longer response to castration therapy exhibit phenotypic and functional patterns associated with high expression of activating receptors and molecules involved in NK cell maturation and degranulation [45].

Sipuleucel-T, a DC-based immunotherapy, has shown improved OS in PCa patients. However, the results have been controversial due to unfavorable outcomes in the control arm. Subsequent DC vaccines have failed to produce significant benefits, and the utility of this immunotherapy strategy remains unproven [79,80].

One study showed that radiotherapy-induced GI or GU toxicity led to an increase in NK cell activity, which was linked to a better prognosis, suggesting that treatment-induced immune activation could benefit patients [97].

Only a limited number of studies have focused on specific NK cell markers, such as NKp30 and NKp46, which have been identified as predictive of TCR and OS in mPCa [45,69]. Furthermore, one 1995 study focused on HNK-1 antigen expression in tumor tissue [33], while another study found no association between IL-2 levels and time to CRPC or OS, although NK cell activity was not directly investigated in this study [98].

#### 2.3.2. NK Cells and Predictive Significance

The role of NK cell activity as a predictor of treatment response, particularly in immunotherapy, has been widely discussed [22]. However, while NK cell activity shows promise as a biomarker, the presence or number of NK cells alone is not yet considered a reliable indicator of treatment outcomes. For instance, Johnson et al. studied patients with non-metastatic PCa who had biochemical recurrence and were treated with anti-tumor DNA vaccination. They found no significant difference in NK cell levels between immune responders and non-responders, suggesting that NK cell quantity alone may not predict treatment success [107].

A few studies have specifically investigated NK cell activity as a predictive biomarker, discussing that NK cell activity levels could potentially predict longer responses to castration therapy [45,98]. However, another study that assessed the role of NK cells in response to DNA vaccination found no significant correlation between NK cell levels and treatment outcomes [107].

A retrospective study of untreated mPCa patients showed that high expression of NKp46 was associated with a better response to castration therapy, with response rates of 39% in patients with high NKp46 expression compared to just 8% in those with low expression [45].

Madan et al. recently investigated the immunologic effects of enzalutamide in non-metastatic hormone sensitive PCa patients. In their study, 38 patients treated with short-course enzalutamide showed an increase in antigen-specific T-cell levels targeting PSA, along with a rise in NK cell numbers and a decrease in myeloid-derived suppressor cells in the blood. However, no significant correlation was found between these immune changes and clinical responses, which may be due to the small sample size [108].

## 3. Microbiome and Immune Interactions in Cancer Including PCa

**Figure 3:**
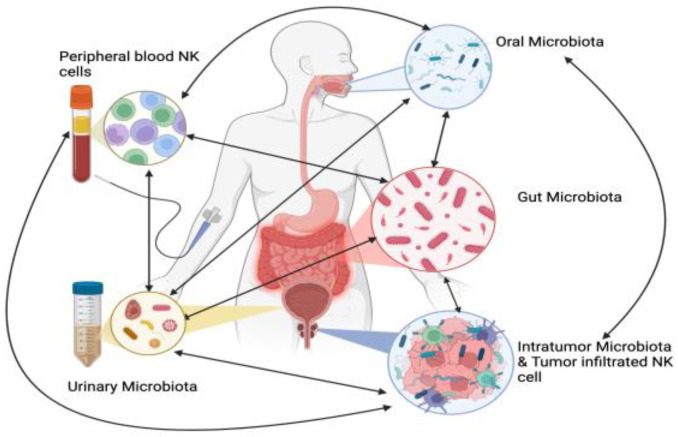
NK-Microbiota Interaction in prostate cancer (PCa). This figure illustrates the intricate interactions between various microbiota (oral, gut, urinary, and tumor-associated) and their influence on NK cells in PCa. The different microbiota communities affect each other: for example, oral microbiota can impact the gut, urinary, and tumor microbiota, while tumor-associated microbiota can also alter the other microbiomes. Dysbiosis, or microbial imbalance, can suppress NK cell function by reducing NK cell frequency, cytotoxicity, and tumor infiltration, impairing immune responses and contributing to tumor initiation, progression, and treatment resistance. On the other hand, a balanced microbiota supports NK cell activity, enhancing tumor surveillance and immune response (Biorender, https://BioRender.com/j36w687 (accessed on 7 February 2025)).

Many studies have examined the link between the microbiome and PCa, exploring its potential as a diagnostic and therapeutic biomarker [109], Figure 3.

The microbiome plays a critical role in metabolism, immune function, and overall health, with disruptions (dysbiosis) linked to various diseases, including cancer. Notably, the oral microbiome has been shown to influence these processes, and when the oral barrier is compromised, microbes can spread through the gut microbiome axis to other parts of the body, potentially contributing to cancer development [110,111].

NK cells play a dual role in both the anti-tumor immune response and defense against microbial infections [112]. Their activity can be influenced by microbiome composition, underscoring the intricate relationship between microbial health and cancer immunity. Further research into the “missing self” hypothesis—where NK cells recognize the absence of MHC molecules—may provide insights into how the microbiome modulates immune surveillance in PCa [113].

The presence of bacterial-derived metabolites in the TME could play a crucial role in modulating immune responses against PCa. Emerging research suggests that specific bacterial metabolites can enhance NK cell cytotoxicity, thereby improving their ability to target and destroy cancer cells. For example, urolithin A, a metabolite derived from dietary polyphenols through gut bacterial metabolism, has been shown to enhance NK cell activation [114]. These metabolites may improve tumor cell elimination in PCa patients.

Findings from the Prostate, Lung, Colorectal, and Ovarian (PLCO) Cancer Screening Trial, which included approximately 155,000 U.S. participants, have provided valuable insights into cancer screening and risk factors [115]. A case-control study from this trial found that elevated levels of certain metabolites, such as choline, carnitine, phenylacetylglutamine (PAGln), and p-cresol sulfate, were associated with a higher risk of lethal PCa [116]. However, associations with other metabolites remain unclear.

The gut microbiome also affects PCa by shaping the TME and regulating gene expression through epigenetic modifications. With a genome over 150 times larger than the human genome, the microbiome has a significant influence on tumor progression via alterations in DNA methylation and histone modifications, which can either promote or suppress cancer growth [117,118].

Bacteria utilize DNA methylation in a process called phase variation, allowing them to switch gene expression and create different bacterial phenotypes. This mechanism can also affect host cells, as bacterial methyltransferases can modify DNA methylation patterns in PCa cells, altering their behavior. Additionally, some bacteria release nucleomodulins, which directly impact the host’s epigenome by modifying DNA and histones, potentially driving tumor progression [119,120].

While this review does not explore microbiome analysis methodologies, which have been extensively covered in previous studies [36], we examine the limited available research on PCa within distinct microbiota communities (oral, gut, urinary, and intra-tumoral) and highlight their potential connections to NK cell activity. However, we do not focus on epigenetics or metabolomics as interactional mechanisms despite their significance and relatively well-studied roles. Instead, we hypothesize that the final phenotype of NK cells, shaped by the microbiome signature, could serve as a surrogate marker for the microbiome’s influence on immune function in PCa. This approach prioritizes the functional outcomes of microbiome-NK cell interactions over the underlying epigenetic and metabolic modifications.

### 3.1. Oral Microbiota

While a retrospective study suggests that reliable oral microbial biomarkers for PCa may not yet exist [121], more recent research has highlighted significant associations between oral microbiota and PCa risk.

A 2020 genome-wide association study (GWAS) conducted in an Asian population, which included 442 PCa cases and 195,745 controls, found strong correlations between PCa and specific bacterial species in the oral cavity. This study identified 27 bacterial species in the tongue, belonging to 17 genera and 14 families, and 42 bacterial species in saliva, spanning 24 genera and 17 families. Notably, nine genera were found in both tongue and saliva samples from PCa patients [35]. Moreover, oral microbial displacement has been linked to the development of PCa [122]. Dysbiosis in the oral microbiota can lead to the colonization of the prostate by oral bacteria, increasing the risk of developing PCa [123].

Interestingly, growing evidence suggests that the host’s microbial ecosystem strongly influences NK cells’ ability to detect and combat cancer [99]. For example, in PCa, the most abundant oral microbiome genus, *Oribacterium*, has been linked to both a reduced number and decreased cytotoxicity of NK cells. [124].

### 3.2. Gut Microbiota

Although the prostate is not physically linked to the gut, lifestyle factors that impact the gut microbiome have been found to influence the pathogenesis of PCa [109]. In prostate cancer, certain bacteria, like Alistipes and Lachnospira, are found in higher levels in the gut, suggesting they may influence cancer progression [125]. Additionally, Reichard et al. demonstrated that certain metabolites are linked to a higher risk of fatal PCa, further linking the microbiome to cancer development [126].

Liss et al. found differences in the microbiome between cancer and non-cancer samples, particularly in Bacteroides and Streptococcus species. They also identified 10 altered metabolic pathways linked to PCa, leading to a new microbiome-based risk factor with a promising diagnostic model (AUC = 0.64, *p* = 0.02) [127].

Zhong et al. found that gut dysbiosis, caused by antibiotics and the overgrowth of Proteobacteria, increased gut permeability and lipopolysaccharide (LPS) levels, which promoted PCa in mice. This suggests Proteobacteria could be a biomarker for PCa progression in humans [128].

Other studies found significant differences in the gut microbiota between PCa cases and controls, specifically the higher relative abundance of Bacteroides and Streptococcus species in cancer cases, while Faecalibacterium prausnitzii and Eubacterium rectale were more abundant in controls. Biologically significant differences were also noted in the relative abundance of genes, pathways, and enzymes associated with these bacteria [126,129].

A mendelian randomization (MR) study identified significant links between 28 gut microbiome taxa and 75 immune cell types with prostate diseases. Notably, the Dorea bacteria may influence immune responses in the prostate, with specific NK cell subsets enhancing its positive effects, potentially lowering the risk of benign prostatic hyperplasia (BPH) [130].

In terms of treatment response, one study showed no significant microbial differences in fecal samples when comparing benign and malignant prostate biopsies [131]. However, Akkermansia muciniphila was found to help regulate changes in the microbiome caused by the drug Abiraterone acetate, suggesting its potential role in modulating treatment responses [132].

There are also distinct differences in the microbiota between hormone-sensitive prostate cancer (HSPC) and castration-resistant prostate cancer (CRPC), highlighting the need for further research. Understanding these differences could help develop strategies to enhance responses to ADT [133]. A preclinical study found that intestinal bacteria and their metabolites, particularly short-chain fatty acids (SCFAs), promoted PCa growth in mouse models [134]. Antibiotic treatment to eliminate the gut microbiota delayed the development of castration resistance, and fecal transplants from CRPC patients or mice made tumors resistant to castration treatment [133,135]. In contrast, transplants from hormone-sensitive patients or the addition of Prevotella stercorea controlled tumor growth, suggesting that the gut microbiota contributes to treatment resistance by producing androgens [135].

*Alistipes* has been shown to have a synergistic effect with PD-1 inhibitors, suggesting that this genus may contribute to elevated PD-1 expression (an exhaustion marker in NK cells), which could influence immunotherapy outcomes. While *Alistipes* serves as a potential predictive marker for immunotherapy response, it is also a negative prognostic marker for disease progression and staging [136].

These findings underscore the complex relationship between the gut microbiome, NK cells and PCa, highlighting its potential role in both cancer progression and treatment response. Further research is needed to fully understand these interactions and identify microbiome-based biomarkers for PCa diagnosis and therapy.

### 3.3. Urinary Microbiota

In the past, urine was believed to be sterile, which limited research on the urinary microbiome. However, recent studies have revealed its potential role in conditions like lower urinary tract symptoms, inflammatory diseases, and cancerous conditions, including PCa. Notably, the act of urination can transfer microbes to the prostate, potentially contributing to prostatic dysbiosis, asymptomatic changes in the prostate environment, and chronic inflammation [137,138,139,140,141].

In PCa patients, specific bacteria like Bacteroidetes and Firmicutes were found to be more abundant, while others, such as Eubacterium, were less common. Escherichia coli was lower in urine but more prevalent in seminal fluid and epididymal prostate secretions (EPS), while Enterococcus levels increased in seminal fluid [142]. A study of the male urinary microbiome discovered that men with PCa had higher levels of pro-inflammatory bacteria and uropathogens in their urinary tract compared to healthy individuals [143]. *Eubacterium* has been shown to enhance NK cell activation when combined with anti-PD-1 therapy, potentially improving immunotherapy efficacy. Specifically, *Eubacterium rectale* enhances the effectiveness of anti-PD-1 treatment in melanoma through L-serine-mediated NK cell activation, suggesting its potential role in modulating immune responses in cancer therapy [144].

A prospective study that examined the link between both urinary and fecal microbiota and PCa diagnosis using 16S rRNA gene analysis found significant differences in the urinary microbiota of PCa patients compared to those without the disease. The study also noted distinct differences in the urinary microbiota of patients with BPH and PCa compared to control subjects [131], emphasizing the need for further research to identify specific microbes that may contribute to prostate disease. Another research identified several novel bacterial species—*Porphyromonas* sp. nov., *Varibaculum* sp. nov., *Peptoniphilus* sp. nov., and *Fenollaria* sp. nov.—in the urine of PCa patients. The presence of certain anaerobic bacteria was linked to a higher risk of cancer and metastasis [145]. These findings support the idea that the urinary microbiome could influence the inflammatory environment of the prostate, potentially impacting NK cell activity and the immune response [142].

Moreover, changes in the urinary microbiome, particularly the decreased abundance of certain bacterial species, have been associated with PCa, especially in patients with a Gleason score of 7 or higher [131]. These alterations highlight the potential of the urinary microbiome as a biomarker for PCa and its role in shaping the prostate’s immune landscape. Although no studies have specifically investigated the interaction between NK cells and the urinary microbiome in PCa, this remains an area of interest for future research.

### 3.4. Intraprostatic/Intratumoral Microbiota

The idea that infections may play a role in PCa was first proposed by Ravich et al. in 1951 [146]. Subsequent research has reinforced this hypothesis, with a meta-analysis of 29 studies linking PCa risk to a history of sexually transmitted infections, such as Mycoplasma genitalium and human papillomavirus (HPV) [147,148]. In addition, chronic inflammation is believed to contribute to PCa development by inducing oxidative stress and genetic mutations [138]. Cavarretta et al. investigated the prostate microbiome and identified bacterial species associated with PCa. However, their study had limitations, as it did not assess the role of inflammation or consider non-bacterial microorganisms, such as mycoplasmas and viruses, that may also influence disease progression [96]. Interestingly, bacterial patterns detected in extracellular vesicles and cancerous tissues have been linked to PCa progression, though the precise role of these bacteria in NK cell suppression and cancer development remains unclear [145].

## 4. Discussion

Understanding the molecular mechanisms underlying NK cell function, microbiota, and their interaction in immune surveillance is critical for advancing PCa therapies. This review provides an overview of the role of NK cells and the microbiome in the management of PCa, highlighting their potential as biomarkers for diagnosis, prognosis, and treatment response. The studies reviewed initially underscore the value of NK cells in guiding clinical decisions and improving patient outcomes as a single biomarker. Initial studies proposed NKA as a promising marker for prognosis and treatment response in PCa [Table 1, Table 2 and Table 3]. Some studies, in particular, highlighted that low NK cell activity levels at diagnosis were associated with shorter TCR and OS [45]. Additionally, one study demonstrated that radiotherapy-induced gastrointestinal or genitourinary toxicity could upregulate NK cell activity, resulting in a better prognosis [97]. Monitoring NK cell function during radiotherapy in patients without radiotherapy-induced side effects could ensure that NK cell activity levels do not decline, thus informing treatment decisions.

NK cell assays in PCa could be utilized to detect microscopic residual disease after prostatectomy, helping to design personalized adjuvant therapies for high-risk patients. For example, low levels of IFN-γ, HNK-1 antigen, alongside high CD56^dim^ to CD56^bright^ NK cell ratios and sMIC may indicate a high risk of undetected microscopic disease [15,33]. Furthermore, some studies investigated NK cell activity as a predictive biomarker. One study found NK cell activity as a potential predictive biomarker to stratify patients likely to have longer castration responses, arguing for therapies aimed at NK cell function augmentation in mPCa patients [45]. Although a small study could not confirm an association between the amount of NK cells and treatment response in PCa patients after DNA vaccination, it was suggested thact different measures of antigen-specific tolerance, or regulation, might help predict the immunological outcome of DNA vaccination [107].

Another study found that in advanced prostate cancer, high NK cell activity in the blood may signal poor response to enzalutamide. This may be due to NK cells failing to infiltrate the tumor, limiting their ability to combat cancer effectively. Instead, these cells stay in circulation, potentially contributing to worse outcomes. Monitoring blood NK cell levels could help identify treatment resistance. This insight may guide personalized treatment strategies for better patient outcomes [149].

However, there are several important limitations to consider. Most of the studies included had relatively small sample sizes, with only a few containing more than 25 participants, which may limit the generalizability of the findings [149]. Additionally, methodological differences across studies—such as the use of outdated technologies, inconsistent patient selection criteria, and variations in statistical analyses—further complicate the interpretation of results. Measuring NK cell activity in the TME presents a significant challenge due to the dynamic and heterogeneous nature of immune responses. While peripheral NK cell assays provide valuable insights, they may not fully reflect NK cell functionality within the TME, where immunosuppressive signals and tumor interactions can modulate NK cell activity. Thus, improved methodologies for assessing NK cell function in the TME, such as advanced imaging and single-cell analyses, are needed.

Although NK cells show promise as biomarkers, it is essential to recognize that NK cells alone may not serve as optimal markers for managing PCa. Integration with other biomarkers, as previously described in other cancers [150], could significantly enhance diagnostic accuracy and prognostication. Recent studies have highlighted the potential role of the microbiome, particularly in the oral, stool, and urinary microbiota, in shaping the prostate’s immune environment. For instance, specific microbial patterns have been linked to PCa risk, inflammation, and disease progression. Microbial dysbiosis could influence the inflammatory microenvironment, subsequently affecting NK cell function.

**Figure 4:**
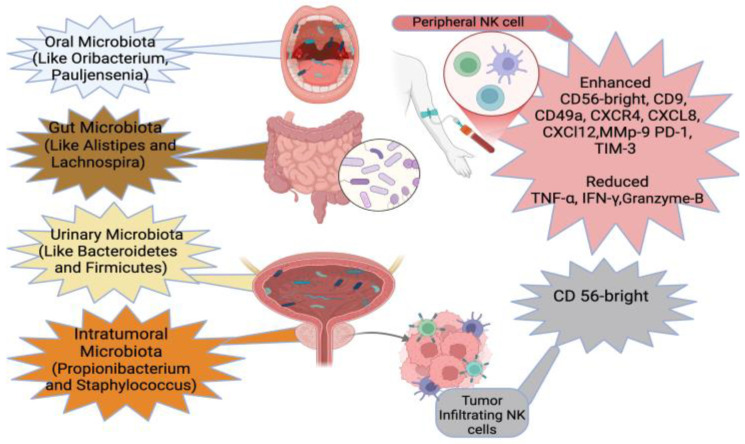
The unified microbiota-NK cell biomarker approach in Prostate Cancer (PCa). In this figure, we illustrate the unified signature for prostate cancer, which integrates the phenotypes of peripheral blood NK cells and tumor-infiltrating NK cells. Additionally, we highlight examples of microbiota signatures associated with prostate cancer, including microbial compositions found in the oral, gut, intraurinary, and intratumoral microbiomes. This unified perspective provides insights into the interplay between immune cell phenotypes and microbial ecosystems in prostate cancer pathophysiology, enhancing tumor surveillance and immune response (Biorender).

Thus, combining NK cell phenotypes with microbiome profiles or utilizing approaches such as the triple NK cell biomarker strategy described earlier [40], which focuses on the interaction between the tumor, TME, and NK cells, could enhance PCa management. This approach targets three mechanisms of NK cell suppression: low proliferation, low toxicity, and low infiltration, offering a more comprehensive and non-invasive diagnostic and therapeutic method. This approach allows for monitoring NKA and the TME using easily accessible biological samples, such as blood, urine, stool, or saliva. By combining NK cell assays with microbiome analysis, it may be possible to track both infiltrating NK cells and circulating NK cell phenotypes as reliable biomarkers for PCa. Non-invasive microbiome testing in oral, urine, or stool samples could be used to assess the tumor’s immune environment, identifying potential therapeutic targets and improving patient outcomes (Figure 4). Once large-scale studies validate the correlation between circulating NK cells and their tumor-infiltrating counterparts, we may be able to rely on circulating NK cells as a surrogate marker for tumor-infiltrating NK cells, combined with microbiome status, for monitoring and managing PCa.

The findings of this review also emphasize the need for larger, more robust clinical trials. A more evidence-based approach, incorporating well-designed prospective cohort studies combining NK cells with microbiome as a linked biomarker, is essential to validate the clinical utility of NK cell assays and microbiome profiling as predictive and prognostic biomarkers in PCa. Additionally, standardizing methodologies for assessing NK cell function in both peripheral blood and the TME will be crucial for ensuring the reliability of findings. Future studies should adhere to established reporting guidelines to ensure their findings are clinically relevant and can be reliably integrated into clinical practice [149]. The limitations of small sample sizes and varying methodologies suggest that a more standardized approach will be key in confirming the role of NK cells and the microbiome in PCa management.

## 5. Conclusions

NK cells have significant potential as complementary biomarkers in the personalized management of PCa. NK cell assays could enhance diagnostic accuracy, prognostication, and treatment monitoring by improving screening, staging, and predicting treatment outcomes. To address the limitations of traditional biopsies, a combinatorial biomarker approach that integrates NK cell phenotypes (from blood samples) with microbiome profiles from easily accessible samples, such as oral, stool, or urine, could serve as a surrogate marker for NK cell activity in the tumor and its TME. This minimally invasive strategy offers a practical alternative to biopsies, enabling more continuous and feasible monitoring of NK cell function in PCa. However, large-scale, prospective studies are needed to validate the clinical utility of this approach and its potential integration into PCa management.

## Figures and Tables

**Table 1:** NK Cells and Pathogenesis.

Year/Reference	No/Population	Sample/Methods	Main Findings
2021/[69]	35/Local, Local advanced PCa 27/Control	PB, Tumor tissue/ Flow cytometry	PCa tumor-associated NK cells show increased pro-inflammatory and pro-angiogenic factors but reduced NKG2D expression, indicating a dysfunctional phenotype.
2018/[68]	10/PCa 10/Control	Tumor tissue, Isolated lymphocyte/ Flow cytometry	PCa lymphocytes have higher miR-224 levels, which protect cancer cells from NK cell attack. HIF-1α upregulates miR-224, inhibiting NCR1/NKp46 and reducing NK cell killing efficiency.
2016/[17]	16/Localized PCa 5/mPCa 20/Normal tissue from patients with PCa	PB, Tumor and normal tissue/Flowcytomery, 51-Cr release	The highly immunosuppressive environment in PCa severely impairs NK cell activity at multiple levels.
2015/[44]	18/Treatment naive PCa, de novo: 10/SCR 8/LCR 10/Control	PM/ Flow cytometry	The low expression of activating receptors and reduced NK cell cytotoxicity contribute to PCa pathogenesis.
2014/[70]	18/CRPC 8/Control	PB, Isolated lymphocyte/Flow cytometry	Prostate tumor-derived exosomes suppress the NKG2D-mediated cytotoxic response.
2013/[14]	51/PCa treatment-naive 54/Healthy control	PB/NK Vue	PCa patients show an increased ratio of CD56^dim^ to CD56^bright^ NK cells compared to healthy controls.
2012/[81]	100/PCa 150/BC	Tumor tissue/Immunohistochemistry	NK cell infiltration in the early stages of PCa is associated with focal disruption of the tumor capsule.
2012/[76]	200/Untreated localized Pca 185/Healthy controls	Blood sample (DNA)/PCR	There is no evidence of a potential role for the KIR gene system in PCa.
2011/[3]	20/Localized, local advanced Pca 20/BPH 20/Healthy controls	PB/Flow cytometry	The percentage of NK cells and their subsets did not vary among the groups examined.
2004/[73]	23/PCa 10/Healthy control	Serum/Cell line Immunohistochemical staining	Advanced cancer is associated with high expression of MICs and low NKA.
1989/[72]	49/Localized and advanced PCa 15/Healthy controls	PB, Cell line /Flowcytometry	Aberrant immune functions occur in the early stages of PCa, with these immune abnormalities worsening in advanced stages.
1989/[71]	7/Localized PCa 6/Advanced PCa 25/Control	PB, Cell line/51-Cr release	MRC in advanced PCa was lower than in localized PCa and the control group.
1987/[15]	10/Localized PCa 49/Advanced PCa 10/BPH 20/Control	PB, Cell line/51-Cr release	Patients with advanced PCa showed significantly lower NKA compared to other groups, a reduction not linked to a lower number of NK cells.
1985/[80]	8/Pca T2N0M0 8/PCa T3-T4NxM0-1 7/Control	PB, Cell line/51-Cr release	Beta interferon-induced NKA enhancement was significantly lower in the periprostatic lymph nodes of PCa patients compared to controls. Additionally, spontaneous NK cell cytotoxicity was greatly reduced in advanced PCa.

Maximal Recycling Capacity (MRC); Long Castration Response (LCR); Peripheral Blood (PB); Prostate Cancer (PCa); Metastatic Prostate Cancer (mPCa); Castration-resistant Prostate Cancer (CRPC); Breast Cancer (BC); Benign Prostatic Hyperplasia (BPH); Natural Killer Activity (NKA); Killer Cell Immunoglobulin-like Receptor (KIR); MHC class I c molecules (MICs).

**Table 3:** NK cell and Prognosis/Prediction.

Year/Reference	No/Population	Sample/Methods	Main Findings
2024/[93]	3365/samples of mPCa 87,551/samples of 44 distinct tumor type	FFPE/ quanTIseq	NK cell infiltration improved outcomes for PCa patients.
2023/[96]	87/mCRPC	PB/NK vue	In late-stage mCRPC patients receiving enzalutamide, higher NKA levels were linked to poorer treatment responses, suggesting that NK cells may negatively affect advanced PCa and could serve as predictors of treatment efficacy.
2020/[97]	24/negative surgical margins 27/positive margins 10/healthy	PB/NK vue	Higher postoperative NKA levels are associated with an increased likelihood of negative surgical margins, indicating a better prognosis.
2018/[98]	19/mPCa 23/mOCa 51/mCRC	PB/NK Vue	Lower NKA levels correlate with reduced response rates and shorter PFS. An increase from low to normal NKA, highlighting its predictive potential.
2017/[99]	22/non metastatic non castrate PCa 16/CRPC	PB/Flow Cytometry	The frequency of NK cells does not differ between immune responders and non-responders following vaccination, suggesting limited predictive value.
2015/[44]	18/Treatment naïve PCa, de novo: 10/SCR 8/LCR 10/Control	PB/Flow Cytometry	NKp30 and NKp46 are significant predictor markers for OS with *p*-values of 0.0018 and 0.0009, respectively. Additionally, TCR shows significance with *p*-values of 0.007, 0.009, and 0.0001, highlighting their prognostic and predictive potential (log-rank test).
2014/[100]	30/mCPC	PB/Flow Cytometry	Higher levels of Tim-3(+) NK cells after vaccination, compared to before vaccination, are associated with longer OS, indicating their prognostic significance.
2014/[101]	72/mPCa before ADT or no longer than 3 months 50/mPCa ADT +/-Risedronate	Serum/ Multiplex electrochemiluminescence	Higher cytokine levels 0.5 months after starting ADT are linked to shorter TCR and OS, suggesting treatment resistance. IL-2 levels, however, do not have prognostic or predictive value as an NK cell activator.
2014/[18]	8/mPCa 36/mBC 30/mCRC	PB/ CellSearch CTC test	High CTCs represent low NKA and a high risk of poor prognosis.
2013/[14]	51/Pca no prior treatment 54/Healthy controls	PB/ NK Vue	A higher CD56^dim^ to CD56^bright^ ratio or lower levels of CD56^bright^ cells are associated with poor prognosis.
2012/[81]	100/PCa 150/BC	Tumor Tissue/ IHC	NK cell infiltration in the early stage PCa is linked to focal disruptions of the tumor capsule and poor prognosis.
2011/[95]	197/PCa, 3DRT 116/T1-T4 WP 81/T1-T4 PO	PB/ Flow cytometry	NK cell numbers are positively linked to bone marrow irradiation (5–25 Gy) and acute/late GU and GI toxicities. This increase in NK cytotoxicity is associated with better prognosis and predictive outcomes.
2009/[23]	35/PCa neoadjuvant ADT + RT 40/Pca RP	Tumor tissue/ IHC	Higher levels of CD56+ NK cells are associated with a lower risk of PCa progression, indicating their potential as prognostic markers.
2004/[73]	23/Localized and advanced PCa 10/Healthy donors	Serum/ ELISA	Higher levels of sMIC are associated with poor prognosis, highlighting its potential prognostic application.
1995 [32]	11/Localized PCa, RP, TURP 41/Advanced, Castration (medical or surgical) 10/BPH	Tumor tissue/51-Cr release	Higher expression of HNK-1 antigen is associated with longer survival and PFS, indicating its prognostic and predictive value.
1989/[71]	7/Localized PCa 6/Advanced PCa 25/Healthy control	PB, cell line K562 /51-Cr release	NKA, Vmax, and MRC levels in advanced PCa are lower than those in localized PCa and control groups, indicating potential prognostic significance.
1980/[102]	6/PCa 7/UBC 24/BC	PB/51-Cr release	NKA is positively associated with prognosis.

Prostate Cancer (PCa); Metastatic Prostate Cancer (mPCa); Breast Cancer (BC); Metastatic Colorectal Cancer (mCRC); Metastatic Ovarian Cancer (mOCa); Urinary Bladder Cancer (UBC); Peripheral Blood (PB); Castration-Resistant Prostate Cancer (CRPC); Natural Killer Activity (NKA); Human Natural killer-1 (HNK-1); Circulating Tumor Cells (CTCs); Soluble MHC class I Chain–related molecules (sMIC); Genitourinary (GU); Gastrointestinal (GI); Radiotherapy (RT); Whole pelvic (WP); Prostate-only (PO); 3-Dimenshional Conformal Radiotherapy (3DRT); Radical prostatectomy (RP); Transurethral Resection of the Prostate (TURP); Overall survival (OS).

## Data Availability

Data sharing is not applicable to this review article as it does not contain original datasets. All relevant information and sources are cited within the manuscript.

## References

[1] Bray F., Laversanne M., Sung H., Ferlay J., Siegel R.L., Soerjomataram I., Jemal A. (2024). Global cancer statistics 2022: GLOBOCAN estimates of incidence and mortality worldwide for 36 cancers in 185 countries. CA Cancer J. Clin..

[2] Hernandez D.J., Nielsen M.E., Han M., Partin A.W. (2007). Contemporary evaluation of the D’amico risk classification of prostate cancer. Urology.

[3] Sotosek S., Sotosek Tokmadzic V., Mrakovcic-Sutic I., Tomas M.I., Dominovic M., Tulic V., Sutic I., Maricic A., Sokolic J., Sustic A. (2011). Comparative study of frequency of different lymphocytes subpopulation in peripheral blood of patients with prostate cancer and benign prostatic hyperplasia. Wien. Klin. Wochenschr..

[4] Neppl-Huber C., Zappa M., Coebergh J.W., Rapiti E., Rachtan J., Holleczek B., Rosso S., Aareleid T., Brenner H., Gondos A. (2012). Changes in incidence, survival and mortality of prostate cancer in Europe and the United States in the PSA era: Additional diagnoses and avoided deaths. Ann. Oncol. Off. J. Eur. Soc. Med. Oncol..

[5] Semjonow A., Brandt B., Oberpenning F., Roth S., Hertle L. (1996). Discordance of assay methods creates pitfalls for the interpretation of prostate-specific antigen values 3–16. Prostate.

[6] Schröder F.H., Carter H.B., Wolters T., van den Bergh R.C., Gosselaar C., Bangma C.H., Roobol M.J. (2008). Early Detection of Prostate Cancer in 2007. Eur. Urol..

[7] Qaseem A., Barry M.J., Denberg T.D., Owens D.K., Shekelle P. (2013). Screening for Prostate Cancer: A Guidance Statement From the Clinical Guidelines Committee of the American College of Physicians. Ann. Intern. Med..

[8] Scarpato K.R., Albertsen P.C. (2016). Prostate-Specific Antigen Screening Guidelines. Prostate Cancer: Science and Clinical Practice.

[9] Hodge K.K., Mcneal J.E., Stamey T.A. (1989). Ultrasound guided transrectal core biopsies of the palpably abnormal prostate. J. Urol..

[10] Djavan B., Ravery V., Zlotta A., Dobronski P., Dobrovits M., Fakhari M., Seitz C., Susani M., Borkowski A., Boccon-Gibod L. (2001). Prospective evaluation of prostate cancer detected on biopsies 1, 2, 3 and 4: When should we stop. J. Urol..

[11] Boesen L. (2019). Magnetic resonance imaging-transrectal ultrasound image fusion guidance of prostate biopsies: Current status, challenges and future perspectives. Scand. J. Urology..

[12] Graham J., Baker M., Macbeth F., Titshall V. (2008). Diagnosis and treatment of prostate cancer: Summary of NICE guidance. Bmj.

[13] Aragon-Ching J.B., Williams K.M., Gulley J.L. (2007). Impact of androgen-deprivation therapy on the immune system: Implications for combination therapy of prostate cancer. Front Biosci..

[14] Koo K.C., Shim D.H., Yang C.M., Lee S.B., Kim S.M., Shin T.Y., Kim K.H., Yoon H.G., Rha K.H., Lee J.M. (2013). Reduction of the CD16-CD56bright NK cell subset precedes NK cell dysfunction in prostate cancer. PLoS ONE.

[15] Choe B.K., Frost P., Morrison M.K., Rose N.R. (1987). Natural killer cell activity of prostatic cancer patients. Cancer Investig..

[16] Hirz T., Mei S., Sarkar H., Kfoury Y., Wu S., Verhoeven B.M., Subtelny A.O., Zlatev D.V., Wszolek M.W., Salari K. (2023). Dissecting the immune suppressive human prostate tumor microenvironment via integrated single-cell and spatial transcriptomic analyses. Nat. Commun..

[17] Pasero C., Gravis G., Guerin M., Granjeaud S., Thomassin-Piana J., Rocchi P., Paciencia-Gros M., Poizat F., Bentobji M., Azario-Cheillan F. (2016). Inherent and Tumor-Driven Immune Tolerance in the Prostate Microenvironment Impairs Natural Killer Cell Antitumor Activity. Cancer Res..

[18] Santos M.F., Mannam V.K., Craft B.S., Puneky L.V., Sheehan N.T., Lewis R.E., Cruse J.M. (2014). Comparative analysis of innate immune system function in metastatic breast, colorectal, and prostate cancerpatients with circulating tumor cells. Exp. Mol. Pathol..

[19] Stovgaard E.S., Nielsen D., Hogdall E., Balslev E. (2017). Triple negative breast cancer–prognostic role of immune-related factors: A systematic review. Acta Oncol..

[20] Abel A.M., Yang C., Thakar M.S., Malarkannan S. (2018). Natural Killer Cells: Development, Maturation, and Clinical Utilization. Front. Immunol..

[21] Hu W., Wang G., Huang D., Sui M., Xu Y. (2019). Cancer Immunotherapy Based on Natural Killer Cells: Current Progress and New Opportunities. Front. Immunol..

[22] Poli A., Michel T., Thérésine M., Andrès E., Hentges F., Zimmer J. (2009). CD56bright natural killer (NK) cells: An important NK cell subset. Immunology.

[23] Gannon P.O., Poisson A.O., Delvoye N., Lapointe R., Mes-Masson A.M., Saad F. (2009). Characterization of the intra-prostatic immune cell infiltration in androgendeprived prostate cancerpatients. J. Immunol. Methods.

[24] Hanna J., Bechtel P., Zhai Y., Youssef F., McLachlan K., Mandelboim O. (2004). Novel Insights on Human NK Cells’ Immunological Modalities Revealed by Gene Expression Profiling. J. Immunol..

[25] Yu C., Young H.A., Ortaldo J.R. (1998). Characterization of cytokine differential induction of STAT complexes in primary human T and NK cells. J. Leukoc. Biol..

[26] Moretta A., Bottino C., Vitale M., Pende D., Cantoni C., Mingari M.C., Biassoni R., Moretta L. (2001). Activating receptors and coreceptors involved in human natural killer cell mediated cytolysis. Annu. Rev. Immunol..

[27] Nausch N., Cerwenka A. (2008). NKG2D ligands in tumor immunity. Oncogene.

[28] Sheppard S., Ferry A., Guedes J., Guerra N. (2018). The Paradoxical Role of NKG2D in Cancer Immunity. Front. Immunol..

[29] Nigro C.L., Macagno M., Sangiolo D., Bertolaccini L., Aglietta M., Merlano M.C. (2019). NK-mediated antibody-dependent cell-mediated cytotoxicity in solid tumors: Biological evidence and clinical perspectives. Ann. Transl. Med..

[30] Kasai M., Leclerc J.C., McVay-Boudreau L., Shen F.W., Cantor H. (1979). Brief Definitive Report Direct Evidence that Natural Killer Cells in Nonimmune Spleen Cell Populations Prevent Tumor Growth In Vivo. J. Exp. Med..

[31] Hood S.P., Foulds G.A., Imrie H., Reeder S., McArdle S.E.B., Khan M., Pockley A.G. (2019). Phenotype and Function of Activated Natural Killer Cells From Patients with Prostate Cancer: Patient-Dependent Responses to Priming and IL-2 Activation. Front. Immunol..

[32] Liu X., Zhan B., Tadao T., Totsuhiro M. (1995). Immunohistochemical study of HNK-1 (leu-7) antigen in prostate cancer and its clinical significance. Chin. Med. J..

[33] Heidegger I., Necchi A., Pircher A., Tsaur I., Marra G., Kasivisvanathan V., Kretschmer A., Mathieu R., Ceci F., van den Bergh R.C.N. (2020). A Systematic Review of the Emerging Role of Immune Checkpoint Inhibitors in Metastatic Castration-resistant Prostate Cancer: Will Combination Strategies Improve Efficacy?. Eur. Urol. Oncol..

[34] Feng K., Ren F., Wang X. (2023). Association between oral microbiome and seven types of cancers in East Asian population: A two-sample Mendelian randomization analysis. Front. Mol. Biosci..

[35] Salachan P.V., Sørensen K.D. (2022). Dysbiotic microbes and how to find them: A review of microbiome profiling in prostate cancer. J. Exp. Clin. Cancer Res..

[36] Ma J., Gnanasekar A., Lee A., Li W.T., Haas M., Wang-Rodriguez J., Chang E.Y., Rajasekaran M., Ongkeko W.M. (2020). Influence of Intratumor Microbiome on Clinical Outcome and Immune Processes in Prostate Cancer. Cancers.

[37] Melaiu O., Lucarini V., Cifaldi L., Fruci D. (2020). Influence of the Tumor Microenvironment on NK Cell Function in Solid Tumors. Front. Immunol..

[38] Jewett A., Kos J., Kaur K., Safaei T., Sutanto C., Chen W., Wong P., Namagerdi A.K., Fang C., Fong Y. (2020). Natural Killer Cells: Diverse Functions in Tumor Immunity and Defects in Pre-neoplastic and Neoplastic Stages of Tumorigenesis. Mol. Ther. Oncolytics.

[39] Fanijavadi S., Thomassen M., Jensen L.H. (2025). Targeting Triple NK Cell Suppression Mechanisms: A Comprehensive Review of Biomarkers in Pancreatic Cancer Therapy. Int. J. Mol. Sci..

[40] Brittenden J. (1996). Natural killer cells and cancer. Cancer.

[41] Zhao S.G., Zhao S.G., Lehrer J., Chang S.L., Das R., Erho N., Liu Y., Sjöström M., Den R.B., Freedland S.J. (2019). The immune landscape of prostate cancer and nomination of PD-L2 as a potential therapeutic target. J. Natl. Cancer Inst..

[42] Liu G., Lu S., Wang X., Page S.T., Higano C.S., Plymate S.R., Greenberg N.M., Sun S., Li Z., Wu J.D. (2013). Perturbation of NK cell peripheral homeostasis accelerates prostate carcinoma metastasis. J. Clin. Investig..

[43] Liu X., Chen Q., Yan J., Wang Y., Zhu C., Chen C., Zhao X., Xu M., Sun Q., Deng R. (2013). MiRNA-296-3p-ICAM-1 axis promotes metastasis of prostate cancer by possible enhancing survival of natural killer cell-resistant circulating tumour cells. Cell Death Dis..

[44] Pasero C., Gravis G., Granjeaud S., Guerin M., Thomassin-Piana J., Rocchi P., Salem N., Walz J., Moretta A., Olive D. (2015). Highly effective NK cells are associated with good prognosis in patients with metastatic prostate cancer. Oncotarget.

[45] Morse M.D., Mcneel D.G. (2010). Prostate cancer patients on androgen deprivation therapy develop persistent changes in adaptive immune responses. Hum. Immunol..

[46] Singh J., Sohal S.S., Ahuja K., Lim A., Duncan H., Thachil T., De Ieso P. (2020). Levels of plasma cytokine in patients undergoing neoadjuvant androgen deprivation therapy and external beam radiation therapy for adenocarcinoma of the prostate. Ann. Transl. Med..

[47] Page S.T., Plymate S.R., Bremner W.J., Matsumoto A.M., Hess D.L., Lin D.W., Amory J.K., Nelson P.S., Wu J.D. (2006). Effect of medical castration on CD4+ CD25+ T cells, CD8+ T cell IFN-*γ* expression, and NK cells: A physiological role for testosterone and/or its metabolites. Am. J. Physiology. Endocrinol. Metab..

[48] Dethlefsen C., Lillelund C., Midtgaard J., Andersen C., Pedersen B.K., Christensen J.F., Hojman P. (2016). Exercise regulates breast cancer cell viability: Systemic training adaptations versus acute exercise responses. Breast Cancer Res. Treat..

[49] Nielsen C.M., White M.J., Goodier M.R., Riley E.M. (2013). Functional significance of CD57 Expression on human NK cells and relevance to disease. Front. Immunol..

[50] Hojan K., Kwiatkowska-Borowczyk E., Leporowska E., Górecki M., Ozga-Majchrzak O., Milecki T., Milecki P. (2016). Physical exercise for functional capacity, blood immune function, fatigue, and quality of life in high-risk prostate cancer patients during radiotherapy: A prospective, randomized clinical study. Eur. J. Phys. Rehabil. Med..

[51] Zheng W., Ling S., Cao Y., Shao C., Sun X. (2024). Combined use of NK cells and radiotherapy in the treatment of solid tumors. Front. Immunol..

[52] Ma Z., Zhang W., Dong B., Xin Z., Ji Y., Su R., Shen K., Pan J., Wang Q., Xue W. (2022). Docetaxel remodels prostate cancer immune microenvironment and enhances checkpoint inhibitor-based immunotherapy. Theranostics.

[53] Khosravi N., Stoner L., Farajivafa V., Hanson E.D. (2019). Exercise training, circulating cytokine levels and immune function in cancer survivors: A meta-analysis. Brain Behav. Immun..

[54] Hanson E.D., Bates L.C., Moertl K., Evans E.S. (2021). Natural Killer Cell Mobilization in Breast and Prostate Cancer Survivors: The Implications of Altered Stress Hormones Following Acute Exercise. Endocrines.

[55] Hanson E.D., Sakkal S., Que S. (2020). Natural killer cell mobilization and egress following acute exercise in men with prostate cancer. Exp. Physiol..

[56] Nieman D.C. (1994). Exercise, infection, and immunity. Int. J. Sports Med..

[57] Maria A.D., Bozzano F., Cantoni C., Moretta L. (2011). Revisiting human natural killer cell subset function revealed cytolytic CD56dimCD16+ NK cells as rapid producers of abundant IFN-*γ* on activation. Proc. Natl. Acad. Sci. USA.

[58] Campbell J.P., Riddell N.E., Burns V.E., Turner M., van Zanten J.J., Drayson M.T., Bosch J.A. (2009). Acute exercise mobilises CD8+ T lymphocytes exhibiting an effector-memory phenotype. Brain Behav. Immun..

[59] Rooney B.V., Bigley A.B., LaVoy E.C., Laughlin M., Pedlar C., Simpson R.J. (2018). Lymphocytes and monocytes egress peripheral blood within minutes after cessation of steady state exercise: A detailed temporal analysis of leukocyte extravasation. Physiol. Behav..

[60] Shephard R.J. (2003). Adhesion molecules, catecholamines and leucocyte redistribution during and following exercise. Sports Med..

[61] Hanson E.D., Sakkal S., Evans W.S., Violet J.A., Battaglini C.L., McConell G.K., Hayes A. (2018). Altered stress hormone response following acute exercise during prostate cancer treatment. Scan-Dinavian J. Med. Sci. Sports.

[62] Galvao D.A., Nosaka K., Taaffe D.R., Peake J., Spry N., Suzuki K., Yamaya K., McGuigan M.R., Kristjanson L.J., Newton R.U. (2008). Endocrine and immune responses to resistance training in prostate cancerpatients. Prostate Cancer Prostatic Dis..

[63] Campbell J.P., Turner J.E. (2018). Debunking the myth of exercise-induced immune suppression: Redefining the impact of exercise on immunological health across the lifespan. Front. Immunol..

[64] Kared H., Martelli S., Ng T.P., Pender S.L., Larbi A. (2016). CD57 in human natural killer cells and T-lymphocytes. Cancer Immunol. Immunother..

[65] Bigley A.B., Rezvani K., Chew C., Sekine T., Pistillo M., Crucian B., Bollard C.M., Simpson R.J. (2014). Acute exercise preferentially redeploys NK-cells with a highly-differentiated phenotype and augments cytotoxicity against lymphoma and multiple myeloma target cells. Brain Behav. Immun..

[66] Vivier E. (2011). Innate or adaptive immunity? The example of natural killer cells. Science.

[67] Wang R., Jaw J.J., Stutzman N.C., Zou Z., Sun P.D. (2012). Natural killer cell-produced IFN-*γ* and TNF-*α* induce target cell cytolysis through up-regulation of ICAM-1. J. Leukoc. Biol..

[68] Chen C., Li S., Xiang L., Mu H., Wang S., Yu K. (2018). HIF-1α induces immune escape of prostate cancer by regulating NCR1/NKp46 signaling through miR-224. Biochem. Biophys. Res. Commun..

[69] Gallazzi M., Baci D., Mortara L., Bosi A., Buono G., Naselli A., Guarneri A., Dehò F., Capogrosso P., Albini A. (2021). Prostate Cancer Peripheral Blood NK Cells Show Enhanced CD9, CD49a, CXCR4, CXCL8, MMP-9 Production and Secrete Monocyte-Recruiting and Polarizing Factors. Front. Immunol..

[70] Lundholm M., Schröder M., Nagaeva O., Baranov V., Widmark A., Mincheva-Nilsson L., Wikström P. (2014). Prostate Tumor-Derived Exosomes Down-Regulate NKG2D Expression on Natural Killer Cells and CD8+ T Cells: Mechanism of Immune Evasion. PLoS ONE.

[71] Marumo K., Ikeuchi K., Baba S., Ueno M., Tazaki H. (1989). Natural killer cell activity and recycling capacity of natural killer cells in patients with carcinoma of the prostate. Keio J. Med..

[72] Lahat N., Alexander B., Dan Levin R., Moskovitz B. (1989). The relationship between clinical stage, natural killer activity and related immunological parameters in adenocarcinoma of the prostate. Cancer Immunol. Immunother..

[73] Wu J.D., Higgins L.M., Steinle A., Cosman D., Haugk K., Plymate S.R. (2004). Prevalent expression of the immunostimulatory MHC class I chain-related molecule is counteracted by shedding in prostate cancer. J. Clin. Investig..

[74] López-Vázquez A., Rodrigo L., Martínez-Borra J., Pérez R., Rodríguez M., Fdez-Morera J.L., Fuentes D., Rodríguez-Rodero S., Gonzáez S., López-Larrea C. (2005). Protective effect of the HLA-Bw4I80 epitope and the killer cell immunoglobulin-like receptor 3DS1 gene against the development of hepatocellular carcinoma in patients with hepatitis C virus infection. J. Infect. Dis..

[75] Middleton D., Vilchez J.R., Cabrera T., Meenagh A., Williams F., Halfpenny I., Maleno I., Ruiz-Cabello F., Lopez-Nevot M.A., Garrido F. (2007). Analysis of KIR gene frequencies in HLA class I characterised bladder, colorectal and laryngeal tumours. Tissue Antigens.

[76] Portela P., Jobim L.F., Salim P.H., Koff W.J., Wilson T.J., Jobim M.R., Schwartsmann G., Roesler R., Jobim M. (2012). Analysis of KIR gene frequencies and HLA class I genotypes in prostate cancer and control group. Int. J. Immunogenet..

[77] Lin S., Chou F.J., Li L., Lin C.Y., Yeh S., Chang C. (2017). Natural killer cells suppress enzalutamide resistance and cell invasion in the castration resistant prostate cancer via targeting the androgen receptor splicing variant 7 (ARv7). Cancer Lett..

[78] Kantoff P.W., Higano C.S., Shore N.D., Berger E.R., Small E.J., Penson D.F., Redfern C.H., Ferrari A.C., Dreicer R., Sims R.B. (2010). Sipuleucel-T immunotherapy for castration-resistant prostate cancer. N. Engl. J. Med..

[79] Jähnisch H., Füssel S., Kiessling A., Wehner R., Zastrow S., Bachmann M., Rieber E.P., Wirth M.P., Schmitz M. (2010). Dendritic cell-based immunotherapy for prostate cancer. Clin. Dev. Immunol..

[80] Wirth M., Schmitz-Dräger B.J., Ackermann R. (1985). Functional properties of natural killer cells in carcinoma of the prostate. J. Urol..

[81] Yuan H., Hsiao Y.H., Zhang Y., Wang J., Yin C., Shen R., Su Y. (2012). Destructive impact of t-lymphocytes, NK and mast cells on basal cell layers: Implications for tumor invasion. BMC Cancer.

[82] Pello O.M., De Pizzol M., Mirolo M., Soucek L., Zammataro L., Amabile A., Doni A., Nebuloni M., Swigart L.B., Evan G.I. (2012). Role of c-MYC in alternative activation of human macrophages and tumor-associated macrophage biology. Blood.

[83] Comito G., Giannoni E., Segura C.P., Barcellos-de-Souza P., Raspollini M.R., Baroni G., Lanciotti M., Serni S., Chiarugi P. (2014). Cancer-associated fibroblasts and M2-polarized macrophages synergize during prostate carcinoma progression. Oncogene.

[84] Dufresne S. (2020). Exercise training improves radiotherapy efficiency in a murine model of prostate cancer. FASEB J. Off. Publ. Fed. Am. Soc. Exp. Biol..

[85] Barkin J., Rodriguez-Suarez R., Betito K. (2017). Association between natural killer cell activity and prostate cancer: A pilot study. Can. J. Urol..

[86] Tarle M., Kraljić I., Kaštelan M. (1993). Comparison between NK cell activity and prostate cancer stage and grade in untreated patients: Correlation with tumor markers and hormonal serotest data. Urol. Res..

[87] Hood S.P., Cosma G., Foulds G.A., Johnson C., Reeder S., McArdle S.E., Khan M.A., Pockley A.G. (2020). Identifying prostate cancer and it’s clinical risk in asymptomatic men using machine learning of high dimensional peripheral blood flow cytometric natural killer cell subset phenotyping data. Elife.

[88] Tae B.S., Jeon B.J., Lee Y.H., Choi H., Park J.Y., Bae J.H. (2020). *Can* natural killer cell activity help screen patients requiring a biopsy for the diagnosis of prostate cancer?. Int. Braz. J. Urol. Off. J. Braz. Soc. Urol..

[89] Vidal A.C., Howard L.E., Wiggins E., De Hoedt A.M., Shiao S.L., Knott S., Taioli E., Fowke J.H., Freedland S.J. (2019). Natural killer cell activity and prostate cancer risk in veteran men undergoing prostate biopsy. Cancer Epidemiol..

[90] Song W., Yu J.W., Jeong B.C., Seo S.I., Jeon S.S., Lee H.M., Choi H.Y., Kang E.S., Jeon H.G. (2018). The clinical usefulness of natural killer cell activity in patients with suspected or diagnosed prostate cancer: An observational cross-sectional study. OncoTargets Ther..

[91] Barkin J., Rodriguez-Suarez R., Betito K. (2017). Immunotherapies can provide opportunities for evaluating human immune responses. Clin Cancer Res..

[92] Coca S., Perez-Piqueras J., Martinez D., Colmenarejo A., Saez M.A., Vallejo C., Martos J.A., Moreno M. (1997). The prognostic significance of intratumoral natural killer cells in patients with colerectal carcinoma. Cancer.

[93] Zorko N.A., Makovec A., Elliott A. (2024). Natural Killer Cell Infiltration in Prostate Cancers Predict Improved Patient Outcomes. Prostate Cancer Prostatic Dis..

[94] Tuong Z.K., Loudon K.W., Berry B., Richoz N., Jones J., Tan X., Nguyen Q., George A., Hori S., Field S. (2021). Resolving the immune landscape of human prostate at a single-cell level in health and cancer. Cell Rep..

[95] Vranova J., Vinakurau S., Richter J., Starec M., Fiserova A., Rosina J. (2011). The evolution of rectal and urinary toxicity and immune response in prostate cancer patients treated with two three-dimensional conformal radiotherapy techniques. Radiat. Oncol..

[96] Zedan A.H., Nedrby L., Volmer L.M., Madsen C.V., Sørensen B.E., Hansen T.F. (2023). Natural killer cell activity in metastatic castration resistant prostate cancerpatients treated with enzalutamide. Sci. Rep..

[97] Lu Y.C., Kuo M.C., Hong J.H., Jaw F.S., Huang C.Y., Cheng J.C., Kung H.N. (2020). Lower postoperative natural killer cell activity is associated with positive surgical margins after radical prostatectomy. J. Formos. Med. Assoc. Taiwan. Yi Zhi.

[98] Hansen T., Nederby L., Zedan A.H., Mejlholm I., Henriksen J.R., Steffensen K.D., Thomsen C.B., Raunkilde L., Jensen L.H., Jakobsen A. (2018). Correlation between natural killer cell activity and treatment effect in patients with disseminated cancer. J. Clin. Oncol..

[99] Johnson L.E., Olson B.M., Mcneel D.G. (2017). Pretreatment antigen-specific immunity and regulation–association with subsequent immune response to anti-tumor DNA vaccination. J. Immunother. Cancer.

[100] Jochems C., Tucker J.A., Tsang K.Y., Madan R.A., Dahut W.L., Liewehr D.J., Steinberg S.M., Gulley J.L., Schlom J. (2014). A combination trial of vaccine plus ipilimumab in metastatic castrationresistant prostate cancer patients: Immune correlates. Cancer Immunol. Immunother..

[101] Sharma J., Gray K.P., Harshman L.C., Evan C., Nakabayashi M., Fichorova R., Rider J., Mucci L., Kantoff P.W., Sweeney C.J. (2014). Elevated IL-8, TNF-*α*, and MCP-1 in men with metastatic prostate cancer starting androgen-deprivation therapy (ADT) are associated with shorter time to castration-resistance and overall survival. Prostate.

[102] Blomgren H., Baral E., Edsmyr F., Strender L.E., Petrini B., Wasserman J. (1980). Natural killer activity in peripheral lymphocyte population following local radiation therapy. Acta Radiologica. Oncol..

[103] Yirga A., Oyekunle T., Howard L.E., De Hoedt A.M., Cooperberg M.R., Kane C.J., Aronson W.J., Terris M.K., Amling C.L., Taioli E. (2021). Monocyte counts and prostate cancer outcomes in white and black men: Results from the SEARCH database. Cancer Causes Control CCC.

[104] Hayashi T., Fujita K., Tanigawa G., Kawashima A., Nagahara A., Ujike T., Uemura M., Takao T., Yamaguchi S., Nonomura N. (2017). Serum monocyte fraction of white blood cells is increased in patients with high Gleason score prostate cancer. Oncotarget.

[105] Shigeta K., Kosaka T., Kitano S., Yasumizu Y., Miyazaki Y., Mizuno R., Shinojima T., Kikuchi E., Miyajima A., Tanoguchi H. (2016). High absolute monocyte count predicts poor clinical outcome in patients with castration-resistant prostate cancer treated with docetaxel chemotherapy. Ann. Surg. Oncol..

[106] Charles A., Thomas R.M. (2023). The Influence of the microbiome on the innate immune microenvironment of solid tumors. Neoplasia.

[107] Madan R.A., Karzai F., Donahue R.N., Al-Harthy M., Bilusic M., Rosner I.I., Singh H., Arlen P.M., Theoret M.R., Marté J.L. (2021). Clinical and immunologic impact of short-course enzalutamide alone and with immunotherapy in non-metastatic castration sensitive prostate cancer. J Immunother. Cancer.

[108] Kim S.J., Park M., Choi A., Yoo S. (2024). Microbiome and Prostate Cancer: Emerging Diagnostic and Therapeutic Opportunities. Pharmaceuticals.

[109] Inamura K. (2023). Oral-Gut Microbiome Crosstalk in Cancer. Cancers.

[110] Park S.Y., Hwang B.O., Lim M., Ok S.H., Lee S.K., Chun K.S., Park K.K., Hu Y., Chung W.Y., Song N.Y. (2021). Oral-Gut Microbiome Axis in Gastrointestinal Disease and Cancer. Cancers.

[111] Chiesa D., Marcenaro E., Sivori S., Carlomagno S., Pesce S., Moretta A. (2014). *Human* NK cell response to pathogens. Semin. Immunol..

[112] Ljunggren H.G., Kärre K. (1990). In search of the “missing self”: MHC molecules and NK cell recognition. Immunol. Today..

[113] Rogovskii V., Murugin V.V., Vorobyev N., Popov S., Sturov N., Krasheninnikov A., Morozov A., Prokhorova M. (2025). Urolithin A increases the natural killer activity of PBMCs in patients with prostate cancer. Front. Pharmacol..

[114] Black A., Huang W.Y., Wright P., Riley T., Mabie J., Mathew S., Ragard L., Hermansen S., Yu K., Pinsky P. (2015). PLCO: Evolution of an Epidemiologic Resource and Opportunities for Future Studies. Rev. Recent. Clin. Trials.

[115] Prakash P., Verma S., Gupta S. (2023). Gut Microbiome and Risk of Lethal Prostate Cancer: Beyond the Boundaries. Cancers.

[116] Reva K., Laranjinha J., Rocha B.S. (2023). Epigenetic Modifications Induced by the Gut Microbiota May Result from What We Eat: Should We Talk about *Precision Diet* in Health and Disease?. Metabolites.

[117] Miya T.V., Marima R., Damane B.P., Ledet E.M., Dlamini Z. (2023). Dissecting Microbiome-Derived SCFAs in Prostate Cancer: Analyzing Gut Microbiota, Racial Disparities, and Epigenetic Mechanisms. Cancers.

[118] Gao Q., Lu S., Wang Y., He L., Wang M., Jia R., Chen S., Zhu D., Liu M., Zhao X. (2023). Bacterial DNA methyltransferase: A key to the epigenetic world with lessons learned from proteobacteria. Front. Microbiol..

[119] Sobhani I., Rotkopf H., Khazaie K. (2020). Bacteria-related changes in host DNA methylation and the risk for CRC. Gut Microbes.

[120] Nearing J.T., Declercq V., Langille M.G. (2023). Investigating the oral microbiome in retrospective and prospective cases of prostate, colon, and breast cancer. Biofilms Microbiomes.

[121] Silva A.P.B.D., Alluri L.S.C., Bissada N.F., Gupta S. (2019). Association between oral pathogens and prostate cancer: Building the relationship. Am. J. Clin. Exp. Urol..

[122] Alluri L.S.C., Paes Batista da Silva A., Verma S., Fu P., Shen D.L., MacLennan G., Gupta S., Bissada N.F. (2021). Presence of Specific Periodontal Pathogens in Prostate Gland Diagnosed with Chronic Inflammation and Adenocarcinoma. Cureus.

[123] Charalambous E.G., Mériaux S.B., Guebels P., Muller C.P., Leenen F.A.D., Elwenspoek M.M.C., Thiele I., Hertel J., Turner J.D. (2021). Early-Life Adversity Leaves Its Imprint on the Oral Microbiome for More Than 20 Years and Is Associated with Long-Term Immune Changes. Int. J. Mol. Sci..

[124] Zha C., Peng Z., Huang K., Tang K., Wang Q., Zhu L., Che B., Li W., Xu S., Huang T. (2023). Potential role of gut microbiota in prostate cancer: Immunity, metabolites, pathways of action?. Front. Oncol..

[125] Reichard C.A., Naelitz B.D., Wang Z., Jia X., Li J., Stampfer M.J., Klein E.A., Hazen S.L., Sharifi N. (2022). Gut Microbiome-Dependent Metabolic Pathways and Risk of Lethal Prostate Cancer: Prospective Analysis of a PLCO Cancer Screening Trial Cohort. Cancer Epidemiol. Biomark. Prev..

[126] Liss M.A., White J.R., Goros M., Gelfond J., Leach R., Johnson-Pais T., Lai Z., Rourke E., Basler J., Ankerst D. (2018). Metabolic Biosynthesis Pathways Identified from Fecal Microbiome Associated with Prostate Cancer. Eur. Urol..

[127] Zhong W., Wu K., Long Z. (2022). Gut dysbiosis promotes prostate cancer progression and docetaxel resistance via activat- ing NF-*κ*B-IL6-STAT3 axis. Microbiome.

[128] Golombos D.M., Ayangbesan A., O’Malley P., Lewicki P., Barlow L., Barbieri C.E., Chan C., DuLong C., Abu-Ali G., Huttenhower C. (2018). The role of gut microbiome in the pathogenesis of prostate cancer: A prospective, pilot study. Urology.

[129] Yue S.-Y. (2024). Causality investigation among gut microbiota, immune cells, and prostate diseases: A Mendelian randomiza- tion study. Front. Microbiol..

[130] Alanee S., El-Zawahry A., Dynda D., Dabaja A., McVary K., Karr M., Braundmeier-Fleming A. (2019). A prospective study to examine the association of the urinary and fecal microbiota with prostate cancer diagnosis after transrectal biopsy of the prostate using 16sRNA gene analysis. Prostate.

[131] Daisley B.A., Chanyi R.M., Abdur-Rashid K., Al K.F., Gibbons S., Chmiel J.A., Wilcox H., Reid G., Anderson A., Dewar M. (2020). Abiraterone acetate preferentially enriches for the gut commensal Akkermansia muciniphila in castrate-resistant prostate cancerpatients. Nat. Commun..

[132] Liu Y., Jiang H. (2020). Compositional differences of gut microbiome in matched hormone-sensitive and castration-resistant prostate cancer. Transl. Androl. Urol..

[133] Matsushita M., Fujita K., Motooka D., Hatano K., Fukae S., Kawamura N., Tomiyama E., Hayashi Y., Banno E., Takao T. (2021). The gut microbiota associated with high-Gleason prostate cancer. Cancer Sci..

[134] Pernigoni N., Zagato E., Calcinotto A., Troiani M., Mestre R.P., Calì B., Attanasio G., Troisi J., Minini M., Mosole S. (2021). Commensal bacteria promote endocrine resistance in prostate cancer through androgen biosynthesis. Science.

[135] Rezasoltani S., Yadegar A., Asadzadeh Aghdaei H., Reza Zali M. (2021). Modulatory effects of gut microbiome in cancer immunotherapy: A novel paradigm for blockade of immune checkpoint inhibitors. Cancer Med..

[136] Yu S.H., Jung S.I. (2022). The potential role of urinary microbiome in benign prostate hyperplasia/lower urinary tract symptoms. Diagnostics.

[137] Sfanos K.S., Yegnasubramanian S., Nelson W.G., Marzo A.M.D. (2018). The inflammatory microenvironment and microbiome in prostate cancer development. Nat. Rev. Urol..

[138] Yow M.A., Tabrizi S.N., Severi G., Bolton D.M., Pedersen J. (2017). Characterisation of microbial communities within aggressive prostate cancer tissues. Infect. Agent. Cancer.

[139] Nelson D.E., Dong Q., Van der Pol B., Toh E., Fan B., Katz B.P., Mi D., Rong R., Weinstock G.M., Sodergren E. (2012). Bacterial communities of the coronal sulcus and distal urethra of adolescent males. PLoS ONE.

[140] Jayalath S., Magana-Arachchi D. (2022). Dysbiosis of the human urinary microbiome and its association to diseases affecting the urinary system. Indian. J. Microbiol..

[141] Yu H., Meng H., Zhou F., Ni X., Shen S., Das U.N. (2015). Urinary microbiota in patients with prostate cancer and benign prostatic hyperplasia. Arch. Med. Sci..

[142] Shrestha E., White J.R., Yu S.H., Kulac I., Ertunc O., De Marzo A.M., Yegnasubramanian S., Mangold L.A., Partin A.W., Sfanos K.S. (2018). Profiling the urinary microbiome in men with positive versus negative biopsies for prostate cancer. J. Urol..

[143] Liu N., Chen L., Yan M., Tao Q., Wu J., Chen J., Chen X., Zhang W., Peng C. (2023). *Eubacterium rectale* Improves the Efficacy of Anti-PD1 Immunotherapy in Melanoma via l -Serine-Mediated NK Cell Activation. Research.

[144] Hurst R., Meader E., Gihawi A., Rallapalli G., Clark J., Kay G.L., Webb M., Manley K., Curley H., Walker H. (2022). Microbiomes of urine and the prostate are linked to human prostate cancer risk groups. Eur. Urol. Oncol..

[145] Ravich A., Ravich R.A. (1951). Prophylaxis of cancer of the prostate, penis, and cervix by circumcision. N. Y. State J. Med..

[146] De Martel C., Franceschi S. (2009). Infections and cancer: Established associations and new hypotheses. Crit. Rev. Oncol. Hematol..

[147] Miyake K., Ohnishi K., Hori S., Nakano A., Nakano R., Yano H., Ohnishi S., Owari T., Morizawa Y., Itami Y. (2019). Mycoplasma genitalium Infection and Chronic Inflammation in Human Prostate Cancer: Detection Using Prostatectomy and Needle Biopsy Specimens. Cell.

[148] Cavarretta R., Ferrarese R., Cazzaniga W., Saita D., Lucianò R., Ceresola E.R., Locatelli I., Visconti L., Lavorgna G., Briganti A. (2017). Microbiome of the Prostate Tumor Microenvironment. Eur. Urol..

[149] Riley R.D., Abrams K.R., Sutton A.J., Lambert P.C., Jones D.R., Heney D., Burchill S.A. (2003). Reporting of prognostic markers: Current problems and development of guidelines for evidence-based practice in the future. Br. J. Cancer.

[150] Fanijavadi S., Jensen L.H. (2025). Dysbiosis-NK Cell Crosstalk in Pancreatic Cancer: Toward a Unified Biomarker Signature for Improved Clinical Outcomes. Int. J. Mol. Sci..

